# Associations between Fetal Symptoms during Pregnancy and Neonatal Clinical Complications with Toxoplasmosis

**DOI:** 10.3390/children11091111

**Published:** 2024-09-11

**Authors:** Nándor Tűzkő, Virág Bartek, Atene Simonyi, Ágnes Harmath, István Szabó, Dezso Peter Virok, Artur Beke

**Affiliations:** 1 Department of Obstetrics and Gynecology, St. Margaret’s Hospital, 1032 Budapest, Hungary; tuzko.nandor@gmail.com; 2 Department of Obstetrics and Gynecology, Semmelweis University, 1085 Budapest, Hungary; bartek.virag@phd.semmelweis.hu (V.B.); atene.simonyi@schiller.ch (A.S.); harmath.agnes@semmelweis.hu (Á.H.); szabo.istvan@semmelweis.hu (I.S.); 3 Department of Medical Microbiology, Albert Szent-Györgyi Health Center, Albert Szent-Györgyi Medical School, University of Szeged, 6720 Szeged, Hungary; virok.dezso.peter@med.u-szeged.hu

**Keywords:** toxoplasmosis, fetal ultrasound, postnatal symptoms, neonatal follow-up

## Abstract

Introduction: Toxoplasmosis is a parasitism transmitted by *Toxoplasma gondii*, part of the TORCH complex, the most prevalent parasitism worldwide. It is asymptomatic in immunocompetent individuals but causes severe infections and developmental abnormalities in pregnant women, mainly affecting the central nervous system and the gastrointestinal system. Methods: In our prospective study, we analyzed cases of recent maternal Toxoplasma infections confirmed by serological testing between 1996 and 2020 at the Department of Obstetrics and Gynecology, Semmelweis University. Amniocentesis, followed by PCR, was performed in cases of recent infection confirmed by serological testing during pregnancy. After birth, a neonatological, microbiological, pediatric neurological and ophthalmological examination and a follow-up was carried out. Results: During the study period, a total of 238 cases of amniotic fluid Toxoplasma PCR testing due to Toxoplasma recent infection were performed. In terms of pregnancies, there were 219 deliveries and seven abortions. Twelve cases had no data available on the outcome of the pregnancy. In total, 133 cases of ultrasound abnormalities were detected during pregnancy, while in 105 cases, no abnormalities were detected on ultrasound examination. During amniocentesis, eight cases of Toxoplasma infection were revealed in amniotic fluid samples by PCR, and in 230 cases, the result was negative. Neonatal follow-up was performed in 139 cases, with no abnormalities during follow-up in 117 cases, and in 22 cases, there was a detectable complication that was likely to be related to Toxoplasma infection. In all 22 cases, amniotic fluid PCR Toxoplasma testing was negative. Conclusions: The most common ultrasound abnormalities involve the nervous system and the gastrointestinal system. In cases of suspicion, it is recommended to perform amniocentesis Toxoplasma PCR testing besides the indirect methods to help the pregnant woman decide whether to carry the pregnancy to term. During follow-up, a multidisciplinary team experienced in pregnancies complicated by toxoplasmosis must carry out the follow-up, care and subsequent development.

## 1. Introduction

Toxoplasmosis is the most common parasitism in the world [1,2], with some estimates suggesting that 60% of the population are carriers [3]. Researchers assume that at least one third of the population has been infected with the infection during their lifetime [4]. Based on estimates, 190,000 new cases of congenital toxoplasmosis occur globally every year [5]. In the statistical data of the European Center for Disease Prevention and Control in 2015, 273 cases of congenital toxoplasmosis are listed in European countries [6].

Toxoplasmosis is part of the TORCH complex (toxoplasmosis, others (syphilis, hepatitis B), rubella, cytomegalovirus and herpes simplex), a parasitism transmitted by *Toxoplasma gondii* [7]. Congenital toxoplasmosis can cause malformations or intrauterine mortality as a result of vertical transmission [8,9]. Pregnant women may contract toxoplasmosis in two ways. More commonly, this is through contact with infected oocysts (consumption of undercooked meat or contact with cat litter) [10,11]. Based on some research, *Toxoplasma gondii* can reach the decidua via the maternal immune cells and leukocytes, or in the decidua itself, entering the immune cells, with the help of which it enters the extravillous cytotrophoblasts [12,13].

Toxoplasma infection can be detected by both direct and indirect methods. The most common indirect method is immunoglobulin detection from maternal blood (IgM) [14].

PCR testing of the amniotic fluid can be used to confirm vertical infection. The first protocol describing the qualitative detection of *Toxoplasma gondii* DNA by PCR dates back to 1989. Burg et al. [15] sequenced and amplified the B1 region of the parasite, which is repeated 35 times in the parasite genome. Since then, other target regions could be sequenced using local protocols, and thus, quantitative measurements can be performed [16].

Timely diagnosis and treatment are essential to prevent serious complications [17].

The severity of congenital toxoplasmosis depends on the week of gestation, and whether the pregnant woman has had the infection before [18]. A treated Toxoplasma infection reduces the chances of both prenatal and postnatal complications. As in all cases, a Toxoplasma infection associated with preterm birth carries a higher risk of subsequent complications. All children with known congenital Toxoplasma infection require close ophthalmologic monitoring until early adulthood [19].

The most common intrauterine fetal malformations are those affecting the nervous system (macrocephaly, hydrocephalus and neural calcification), small for gestational age/intrauterine growth restriction (SGA/IUGR) and hepatosplenomegaly [20,21,22]. Ultrasound signs that may alert to intrauterine infection include intracranial calcification, ventriculomegaly, hydrocephalus, hepatosplenomegaly with or without calcification involving the gastrointestinal system, and echogenic bowels. In addition, ascites, pericardial effusion and hydrops or polyhydramnios may be present. SGA/IUGR is also frequently observed [23].

The classic congenital toxoplasmosis “triad” of choriorenitis, hydrocephalus and neurological calcification occurs in cases where the pregnant woman acquires the primary infection predominantly in the first trimester and receives no further treatment. However, in 85% of the cases, no abnormality is found by routine neonatal investigations. In a study in 1994, Guerina et al. found that 40% of the confirmed cases of intrauterine infection were found to be free of abnormalities on routine neonatal examination, but later, ophthalmological or neurological abnormalities were revealed. Therefore, early diagnosis of these subclinical abnormalities could reduce the extent of subsequent damage [24].

Accordingly, neonatal and late complications most frequently affect the nervous system, and the proportion of children with special educational needs and late speech and motor development is higher in this group. Ophthalmological complications, early visual impairment and hearing impairment are also observed. Toxoplasma choriorenitis affects approximately 21,000 people worldwide each year [25].

Neonatal screening should be systematic and involve co-specialists. Blood samples should be sent for serological testing from both the newborn and the mother as soon as possible after birth.

Newborn IgM and IgA levels should be checked at the age of 3 months and IgG levels by the age of 1 year to screen for late seroconversion. By the age of 1 year, all maternally derived Toxoplasma-specific IgG had disappeared from the infant. In addition to serological tests, the newborn’s complete blood count and liver and kidney function values should also be checked.

The neonate should also be referred to a pediatric ophthalmologist, and lifelong ophthalmic follow-up is recommended for the affected patients. More frequent hearing tests are also advised.

For newborns with organ damage, treatment should be started as soon as possible. There is currently no clear standard of care, but in most countries, pyrimethamine, sulfadiazine and folic acid are recommended in combination up to 1 year of age. Both treated and suspected cases of infection should be kept under close neonatal and then pediatric check-ups [23].

## 2. Materials and Methods

In our prospective study, we analyzed cases of recent maternal Toxoplasma infections confirmed by serological testing at the Department of Obstetrics and Gynecology, Semmelweis University, Hungary, between 1996 and 2020.

We included in the study those pregnant women who applied for genetic counseling at our department and who were confirmed to be infected with Toxoplasma by serological testing and who requested amniocentesis. Those who had a miscarriage before amniocentesis or did not request amniocentesis were excluded from the study.

The serological tests (IgG and IgM determination) were performed at the Central Laboratory of Semmelweis University in accordance with international recommendations, and IgA determination was also performed in cases of acute infection.

In recent infections detected during serological testing, genetic counseling was provided, where the couple was informed in detail, additional ultrasound examinations were performed if necessary and amniocentesis was offered to detect fetal involvement.

The ultrasound examinations were performed in the Ultrasound Laboratory, Department of the Department of Obstetrics and Gynecology, Baross Street, using Medison Sonoace X8 (Medison Co., Ltd., Seoul, Republic of Korea), Samsung Medison UGEO H60 (Samsung Medison Co., Ltd., Seoul, Republic of Korea), Samsung Medison WS80A (Samsung Medison Co., Ltd., Seoul, Republic of Korea) and Philips^®^ HD 11XE (Philips Ultasound, Amsterdam, The Netherlands) ultrasound equipment. The examinations were performed in accordance with the professional protocols developed by the Hungarian Obstetric–Gynecological Ultrasound Society (Obstetric pregnancy transabdominal ultrasound examination—10 February 2003; Fetal echocardiography—10 February 2003; Ultrasound examinations recommended during pregnancy—10 February 2003) and the current Health Professional Guidelines (On diagnostic and basic ultrasound screening examinations in early pregnancy, 2016). Significant fetal anomalies include fetal cranial abnormalities (ventriculomegaly, cerebral calcification, ventricular dilatation III and IV and microcephaly), subcutaneous edema (NT, hydrops, anasarca and hygroma), cardiac and thoracic abnormalities (pericardial effusion, pleural effusion and chest tightness), abdominal anomalies (echodense bowels, distended bowels, echodense liver, perihepatic fluid, ascites and pyelectasia) and placental anomalies (cystic placental abruptions, thickened placenta, premature calcification and amniotic bands).

In cases of ultrasound signs suggestive of infection, genetic counseling was provided, during which the expectant mother or couple was informed in detail about the possible risks and complications of the infection. If the serological result was positive or inconclusive, or if a new or worsening lesion was seen on a check-up ultrasound examination, amniocentesis was recommended to detect possible intrauterine transmission.

After amniocentesis the amniotic fluid sample was stored at 4–8 °C, and after 1–2 h we performed DNA isolation. After DNA isolation we stored the samples at −80 °C. DNA isolation from amniotic fluid was performed at the Genetics Laboratory of the Department of Obstetrics and Genetics, Semmelweis University, using the silica gel adsorption technique (High Pure PCR Template Isolation Kit, Roche, Mannheim, Germany). For the positive control, T. gondii (RH strain) DNA was extracted from purified parasites obtained from the ascites fluid of intraperitoneally inoculated mice. Tenfold dilution with distilled water was used to obtain DNA concentrations corresponding to 108–102 parasites. Fluorescent PCR and DNA fragment analysis were used for the assay. For amplification, a 1 μL DNA sample was used. Toxo B22, TET-5′-AAC GGG CGA GTA GCA CCT GAG GAG A-3′ and Toxo B23, 5′-TGG GTC TAC GTC GAT GGC ATG ACA AC-3′ primers were used. The PCR reaction mixture contained 2.5 μL PCR buffer, 2.5 μL 10 mM dNTP, 2.5 μL 25 mM MgCI2 and 0.15 U AtaqGold polymerase in a final volume of 25 μL. The initial incubation took 10 min at 95 °C, followed by denaturation (95 °C, 30 s), tempering (55 °C, 45 s), extension (72 °C, 45 s) for 36 cycles and finally, an incubation at 72 °C for 30 min. Four microliters of F-PCR product were mixed with 20 microliters of formamide (Sigma-Aldrich, St. Louis, MO, USA) and one microliter of Prism GeneScan-500 TAMRA internal standard (PE Applied Biosystems, Warrington, UK). The mixture was then denatured at 95 °C for 3 min and cooled to 4 °C for 5 min. Electrophoresis was performed on an ABI 310 Genetic Analyser using POP4 gel (PE Applied Biosystems, Foster City, CA, USA). The results were analyzed using GeneScan Analysis software 3.1 (PE).

After birth, a neonatological, microbiological, pediatric neurological and ophthalmological examination was carried out and follow-up was carried out. In the perinatal period, after the maternal and newborn serological examination, a cranial ultrasound is performed and an ophthalmological examination is performed. In positive cases, medication is given. Children with Toxoplasma infection undergo regular neurological and ophthalmological control and follow-up.

The aim of our study was to investigate which ultrasound abnormalities are most commonly associated with active Toxoplasma infection and the long-term complications of the infection. We aimed to investigate whether there is an association between expected complications and prenatal abnormalities.

The differences in categorical variables were assessed using Fisher’s exact test. Statistical analysis was conducted using Stata Statistical Software (version 13.0, StataCorp, College Station, TX, USA), with statistical significance set at *p* < 0.05.

## 3. Results

At the Department of Obstetrics and Gynecology, Baross Street, Semmelweis University, Hungary, between 1996 and 2020, 238 cases of Toxoplasma infection were studied, where Toxoplasma infection was confirmed by serological testing, and amniotic fluid Toxoplasma PCR was performed. The mean age of the mothers examined was 29.47 ± 5 years.

In terms of the outcome of pregnancies, there were 219 deliveries and seven terminations of pregnancy (TOP).

In 230 of the 238 cases, the presence of Toxoplasma DNA in the amniotic fluid was not detected by PCR. TOP was chosen in only two of these cases (0.8%). We were able to detect Toxoplasma DNA in the amniotic fluid in a total of eight cases, and TOP occurred in five of these cases (62%).

**Figure 1:**
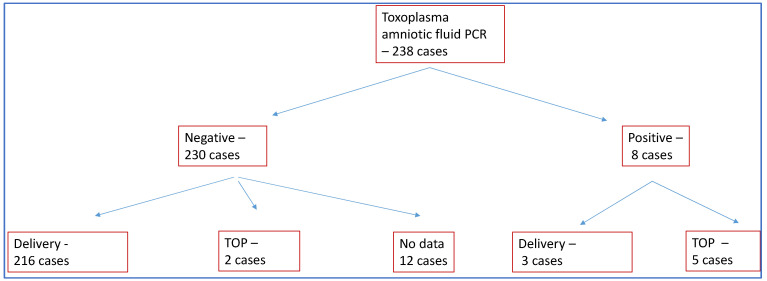
Patients participating in the study. TOP: termination of pregnancy.

In 12 cases, no data were available on the outcome of the pregnancy, with the pregnant woman receiving further care at another institution (Figure 1). A total of 25 cases of induced labor occurred. Birth was given at an average of 38.91 ± 1.94 weeks of gestation, and abortions at an average of 22 ± 1.41 weeks of gestation. Of the 219 deliveries, 132 were spontaneous vaginal deliveries, 69 were cesarean deliveries and in 18 cases, no information was available on the mode of delivery due to care being provided at another institution. One case of intrauterine death occurred. The proportion of preterm births was 8%. There were two IVF-ET, two ICSI pregnancies and five twin pregnancies during the study period.

### 3.1. Processing of Ultrasound Results

**Figure 2:**
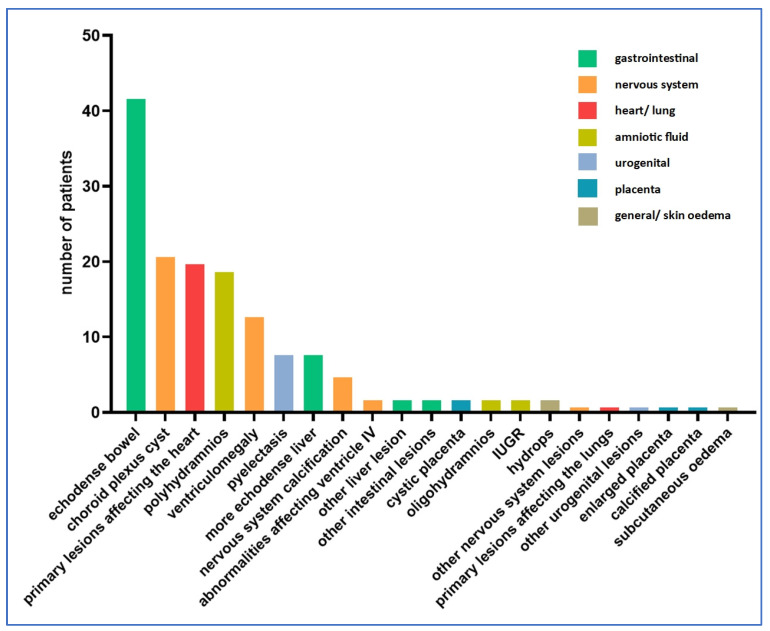
Distribution of ultrasound abnormalities (n = 105 cases).

In total, 133 cases of ultrasound abnormalities were detected during pregnancy, while 105 cases had no abnormalities detected during ultrasound examinations. Figure 2 shows the distribution of ultrasound abnormalities.

Among the amniotic fluid abnormalities, polyhydramnios was dominant, being present in 19 cases (14%). Oligohydramnios was detected in two cases (1%). Fetal growth retardation was also detected in two cases (1%).

The following is a summary of ultrasound findings by organ systems. The total numbers of data for each case shown are projected to the 133 positive findings.

Fetal edematous abnormalities included two cases of fetal hydrops (1.5%) and one case of subcutaneous edema (0.8%). No thicker nuchal fold was observed in any of the cases.

In our study, the most commonly affected organ system was the craniospinal one. A total of 42 abnormalities involving the nervous system were described in 38 fetuses (29%) (four cases with two nervous system anomalies simultaneously), the most common being choroid plexus cyst (21 cases, 15.8%) and ventriculomegaly (13 cases, 9.8%).

Nervous system calcification was present in five cases (3.8%). A lesion involving ventricle IV was described in two cases (1.5%). In the sample studied, ventricle III, the posterior fossa and the cisterna magna were not involved in any of the cases.

We examined the lesions detected in the thoracic organs. In total, 21 cases of ultrasound abnormalities were detected (15.8%). Among the thoracic organs, the heart was most frequently involved (20 cases, 15%). One case of pulmonary abnormality was described (0.8%).

Abnormalities affecting the urogenital system occurred in nine cases (6.8%). The most common of the abnormalities affecting the urogenital system was pyelectasia, with eight cases (6%).

Abnormalities of the gastrointestinal system were also common (52 cases, 39.1%). Echogenicity enhancement may often indicate a possible Toxoplasma infection, with eight cases of echodense liver (6%) and 42 cases of echodense bowels (31.6%). Further liver lesions were detected in two cases, and further intestinal lesions were also identified in two cases (1.5% each).

Abnormalities affecting the placenta were less frequent, with a total of four cases where abnormalities concerning the placenta (4.5%) were identified by ultrasonography. One case of thickened placenta (0.8%), two cases of cystic placental abruptions (1.5%) and one case of calcified placenta (0.8%) were detected.

Two cases of other lesions not classified in any of the above tables were described (1.5%). Two cases of lesions involving more than one organ system were found. Both cases showed intracranial calcification and an echodense liver. In 67 cases, ultrasound follow-up revealed worsening of the lesion.

### 3.2. Processing of PCR Results

Serological testing of maternal blood showed IgG positivity in 234 out of the 238 cases with positive IgM, whereas four cases had borderline (equivocal) IgM.

From amniotic fluid sampling, eight cases of Toxoplasma were detected by PCR, and negative results were obtained in 230 cases. Of the positive PCR samples, five pregnancies were aborted, and three pregnancies were carried to term. In all three of the latter cases, a mature baby was born.

Although the positive amniocentesis PCR rate was significantly lower, it can be seen that when the amniocentesis sample was negative, a higher proportion of pregnant women chose to be carried to term (Table 1).

### 3.3. Neonatological Follow-Up

**Figure 3:**
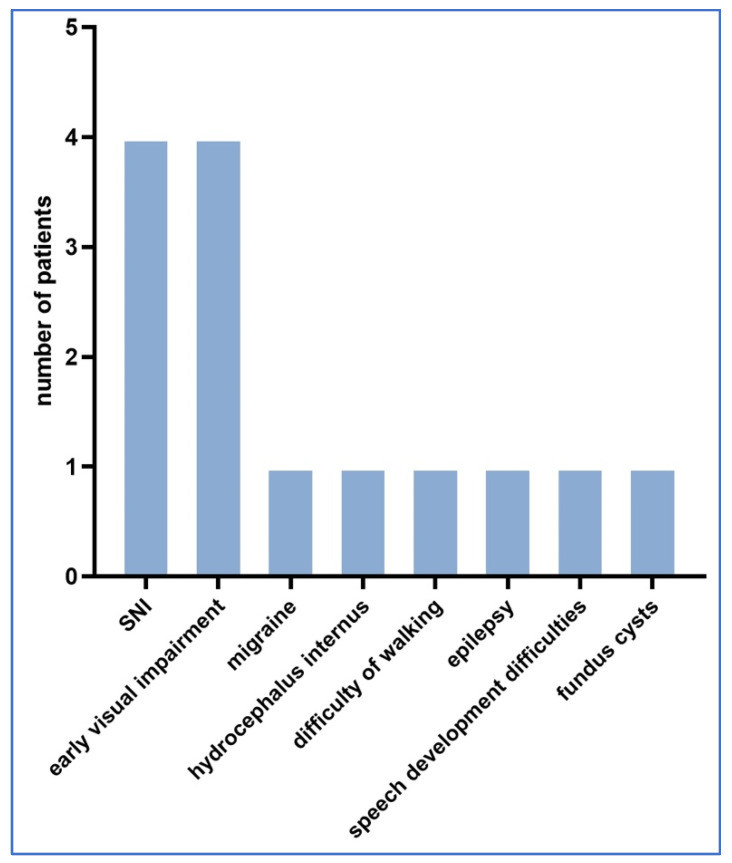
Distribution of neurological and other abnormalities detected during follow-up.

Neonatological follow-up was performed in 139 cases and 117 cases had no abnormalities during the follow-up, whereas 22 cases had detectable complications probably related to Toxoplasma infection. Figure 3 shows the distribution of neurological and other abnormalities detected during follow-up.

In all 22 cases, amniotic fluid PCR Toxoplasma testing was negative. Nine cases had neurological complications. In the follow-up cases studied, there were five cases with ophthalmological complications.

Other complications included: three cases of frequent ear infections, one child with no hearing in the left ear and four children who developed jaundice after birth but healed after blue light treatment, with no permanent sequelae. No postnatal deaths occurred in any of the cases.

A chi-square test was used to investigate whether there was a correlation between prenatal factors and subsequent complications. The results are presented in Table 2 below.

As it can be seen from the above table, no significant correlation with the expected outcome could be demonstrated in any of the cases. However, the clinical relevance of this is questionable, as the rate of complications was low.

We also investigated whether seropositivity associated with ultrasound abnormalities could predict the occurrence of neonatal neurological and ophthalmological complications. The results are illustrated in Table 3 and Table 4. In cases with fetal subcutaneous oedema there was significant association with neonatal neurological complications.

## 4. Discussion

### 4.1. Abnormalities Detected on Ultrasound Examination

The diagnosis and treatment options for Toxoplasma infections have been addressed in a number of publications. Codaccioni et al. [26] have evaluated the ultrasound findings of 88 pregnant women with confirmed Toxoplasma infection. In 45 (51.1%) cases, there was one or more craniocerebral abnormalities, in eight (9.1%) other organ systems were involved. The most common intracranial abnormalities were calcification (60 cases) and ventriculomegaly (44 cases), which worsened with advancing pregnancy. In our study, intracranial abnormalities were also very common (29%), although we found a lower number of cases than published in the literature. Likewise, the most common intracranial abnormalities were calcification and ventriculomegaly, the latter being more common in our study (ventriculomegaly n = 13, calcification n = 5). We did not investigate the progression of ventriculomegaly.

The most common extracranial abnormalities were liver abnormalities (mainly hepatomegaly, 14 cases), followed by increased intestinal echogenicity (11 cases). In the period processed, the rate of increased intestinal echogenicity was much higher in our study (31.6% of cases) than in the report of Codaccioni et al. [26].

Hohlfeld et al. have studied 89 pregnancies with confirmed Toxoplasma infection. The most common abnormality (n = 25) was ventriculomegaly, followed by intracranial calcification (n = 6). Their data are virtually identical with our data, but in percentage, these were lower in our study. Placental abnormalities were also examined, in which 11 cases were described as thickening and two cases as increased calcification. In our study, there was only one of each of these cases [27].

A comparison of these published results and our own results is summarized in Table 5 below.

The reason for the differences in the results may be due to that Toxoplasma infection causes a wide variety of developmental abnormalities, thus leading to a corresponding variety of ultrasound findings. We may conclude that although the percentage of variation in each organ system varies within a wide range, the results processed in our study are proportionally in line with the literature.

### 4.2. PCR Tests from Amniotic Fluid

In 2016, De Oliveira Azevedo [28] conducted a comprehensive meta-analysis of studies in which PCR testing was performed on amniotic fluid samples from pregnant patients to detect Toxoplasma. A total of 4171 cases from 20 studies were analyzed.

Three studies investigated the gestational age at acute infection, ranging from 15.6 to 19.6 weeks, which is also consistent with our results (18.8 ± 2.3 weeks of gestation).

The results reflect that the sensitivity of both IgM and PCR screening increases with advancing gestational age; nonetheless, there are no significant statistical results. The reviewed literature all points in this direction, and our study also confirmed this.

According to current professional recommendations, amniocentesis was only performed in the second trimester, so seroconversion rates could only be investigated then. However, based on the literature data, the sensitivity of amniocentesis in the first trimester is inherently much lower, as placental permeability is low and few fetal cells can be recovered from the amniotic fluid.

Overall, both the meta-analysis and our study confirm that the detection of *Toxoplasma gondii* DNA by PCR from amniotic fluid is currently the most reliable, rapid and low-invasive method for the detection of intrauterine Toxoplasma infection, even with the given limitations [28].

### 4.3. Follow-Up Examination of the Newborn

Caceres et al. have investigated neurological complications. While most infected newborns have developed no complications, among those with intracranial involvement, 8% to 12% of the cases presented with intracerebral calcifications, 4% to 30% with hydrocephalus and 12% to 15% with choriorenitis. Subsequent neurological involvement has been present in 12% of cases, proportionally consistent with our data [29].

In a retrospective study, Reynolds et al. have investigated the presence and management of congenital macula laesio in their patients. In their practice, nine children required ophthalmic intervention, five of which had macular scarring. On average, the complaints occurred before the age of 3 years. All patients improved with ophthalmic intervention, regardless of the severity and size of the macular lesion. Their professional recommendation is that ophthalmic intervention for Toxoplasma infection with ocular complications can greatly improve the quality of life and is therefore recommended [30].

Ferreira et al. [31] have published a literature review on the impact of congenital Toxoplasma infection on hearing. This paper suggests that hearing impairment caused by Toxoplasma infection has both peripheral and central components. Research has also found an association between congenital Toxoplasma infection and mild to moderate hearing loss; however, these studies did not investigate whether risk factors for hearing impairment other than congenital infection were present in the affected patients (e.g., prematurity, ototoxic medications, etc.).

In our study, we found one case of hearing impairment and one case of a recurrent ear infection during follow-up.

As the review of the literature has not yet described a parallel between recurrent otitis media and congenital Toxoplasma infection; it is likely to be an independent factor [31].

There were 230 negative PCR results, but there were also 22 cases where complications still arose despite negative PCR findings. The study draws attention to the limitations of PCR tests. The PCR tests were performed at a specific time interval, during a certain period of pregnancy, and were not repeated at a later stage due to the risks of amniocentesis. Therefore, it could happen that the PCR result was negative at the time of the test, but later it could become positive, but this was no longer detected.

In 12 cases, no information was available about the post-natal examinations. The reason for this was that these births took place in another institution and no data on the newborns were available after the birth. This small number of cases slightly limits the results compared to all cases.

## 5. Conclusions

The most common ultrasound abnormality affects the nervous system and the gastrointestinal system. In case of suspicion, in addition to indirect methods, it is recommended to carry out a PCR test, helping the pregnant woman to make a decision regarding pregnancy. We could not prove a significant correlation between positive PCR and subsequent complications. Regarding the severity and appearance of complications, we did not find any prenatal factors with which we could prove a significant correlation.

While ultrasound and PCR testing remain indispensable tools in managing toxoplasmosis during pregnancy, further research is needed to identify additional factors that may contribute to the variability in clinical outcomes. During follow-up, a multidisciplinary team experienced in pregnancies complicated by toxoplasmosis must carry out the follow-up, care and subsequent development.

## Figures and Tables

**Table 1:** Outcome of pregnancies with recent Toxoplasma infection as a function of PCR results (n = 238).

	Amniotic Fluid PCR Positive n = 8	Amniotic Fluid PCR Negative n = 230
Delivery	3 (38%)	216 (93.9%)
Abortion	5 (62%)	2 (0.8%)
Stillbirth		1 (0.5%)

**Table 2:** Associations between prenatal factors and subsequent complications.

Prenatal Factors	Complications (n = 22)	No Complications (n = 117)	Total	*p*
Age over 37 years	1	13	14	0.407890
PCR positive	0	2	2	0.557217
Preterm birth	2	7	9	0.495630
Sex of the fetus (boy)	13	52	65	0.076257
Twin pregnancy	1	2	3	0.348785
IVF (ET or ICSI)	0	4	4	0.402803
Infection in the first trimester of pregnancy	4	13	17	0.257281

IVF: in vitro fertilization; ET: embryo transfer; ICSI: intracytoplasmic sperm injection.

**Table 3:** Association between neonatal neurological complications and fetal ultrasound malformations.

		Fetal Malformation on Ultrasound (218 Livebirths)					
		Negative		Positive		Total	Fisher’s Exact Test
		Case	%	Case	%		
Neonatal neurological complications							
		Subcutan oedema					
Negative	case	210	100.00%	0	0.00%	210	
	%	96.77%		0.00%			
Positive	case	7	87.50%	1	12.50%	8	
	%	3.23%		100%			
Total		217	99.54%	1	0.46%	218	0.037
		Craniospinal malformations					
Negative	case	181	86.19%	29	13.81%	210	
	%	96.28%		96.67%			
Positive	case	7	87.50%	1	12.50%	8	
	%	3.72%		3.33%			
Total		188	86.24%	30	13.76%	218	0.697
		Cardiovascular malformations					
Negative	case	192	91.43%	18	8.57%	210	
	%	96.00%		100%			
Positive	case	8	100.00%	0	0.00%	8	
	%	4.00%		0%			
Total		200	91.74%	18	8.26%	218	0.697
		Pulmonary malformations					
Negative	case	209	99.52%	1	0.48%	210	
	%	96.31%		0.00%			
Positive	case	8	100.00%	0	0.00%	8	
	%	3.23%		0.00%			
Total		217	99.54%	1	0.46%	218	0.963
		Urogenital malformations					
Negative	case	203	96.67%	7	3.33%	210	
	%	96.21%		100.00%			
Positive	case	8	100.00%	0	0.00%	8	
	%	3.79%		0.00%			
Total		211	96.79%	7	3.21%	218	0.767
		Hepatic malformations					
Negative	case	203	96.67%	7	3.33%	210	
	%	96.21%		100.00%			
Positive	case	8	100.00%	0	0.00%	8	
	%	3.79%		0.00%			
Total		211	96.79%	7	3.21%	218	0.767
		Gastrointestinal malformations					
Negative	case	170	80.95%	40	19.05%	210	
	%	95.51%		100.00%			
Positive	case	8	100.00%	0	0.00%	8	
	%	4.49%		0.00%			
Total		178	81.65%	40	18.35%	218	0.192

**Table 4:** Association between ophthalmological neurological complications and fetal ultrasound malformations.

		Fetal Malformation on Ultrasound (218 Livebirths)					
		Negative		Positive		Total	Fisher’s Exact Test
		Case	%	Case	%		
Neonatal ophthalmological complication							
		Subcutan oedema					
Negative	case	209	99.05%	2	0.95%	211	
	%	96.76%		100.00%			
Positive	case	7	100.00%	0	0.00%	7	
	%	3.24%		0.00%			
Total		216	99.08%	2	0.92%	218	0.937
		Craniospinal malformations					
Negative	case	181	85.78%	30	14.22%	211	
	%	96.28%		100.00%			
Positive	case	7	100.00%	0	0.00%	7	
	%	3.72%		0.00%			
Total		188	86.24%	30	13.76%	218	0.349
		Cardiovascular malformations					
Negative	case	193	91.47%	18	8.53%	211	
	%	96.50%		100.00%			
Positive	case	7	100.00%	0	0.00%	7	
	%	3.50%		0.00%			
Total		200	91.74%	18	8.26%	218	0.542
		Pulmonary malformations					
Negative	case	210	99.53%	1	0.47%	211	
	%	96.77%		100.00%			
Positive	case	7	100.00%	0	0.00%	7	
	%	3.23%		0.00%			
Total		217	99.54%	1	0.46%	218	0.968
		Urogenital malformations					
Negative	case	204	96.68%	7	3.32%	211	
	%	96.68%		100.00%			
Positive	case	7	100.00%	0	0.00%	7	
	%	3.32%		0.00%			
Total		211	96.79%	7	3.21%	218	0.793
		Hepatic malformations					
Negative	case	205	97.16%	6	2.84%	211	
	%	97.16%		85.71%			
Positive	case	6	85.71%	1	14.29%	7	
	%	2.84%		14.29%			
Total		211	96.79%	7	3.21%	218	0.207
		Gastrointestinal malformations					
Negative	case	172	81.52%	39	18.48%	211	
	%	96.63%		97.50%			
Positive	case	6	85.71%	1	14.29%	7	
	%	3.37%		2.50%			
Total		178	81.65%	40	18.35%	218	0.622

**Table 5:** Literature comparison between various studies on prenatally detected ultrasound abnormalities.

	Codaccioni et al. [26]	Hohlfeld et al. [27]	Results of the Present Study
Number of cases	88	89	133
Intracranial abnormalities			
Ventriculomegaly	44 (50%)	25 (28%)	13 (9.8%)
Abnormalities affecting ventricle IV	not tested	not tested	2 (1.5%)
Choroid plexus cyst	not tested	not tested	21 (15.8%)
Nervous system calcification	5 (5.7%)	6 (6.7%)	5 (3.8%)
Abnormalities affecting the gastrointestinal system			
Echodense liver	not tested	4 (4.5%)	8 (6%)
Echodense intestines	11 (12.5%)	not tested	42 (36.6%)

## Data Availability

The datasets used and/or analyzed during the current study are available from the corresponding author on reasonable request due to privacy and ethical reasons.
